# Spectral features of the tunneling-induced transparency and the Autler-Townes doublet and triplet in a triple quantum dot

**DOI:** 10.1038/s41598-018-21221-3

**Published:** 2018-02-15

**Authors:** Xiao-Qing Luo, Zeng-Zhao Li, Jun Jing, Wei Xiong, Tie-Fu Li, Ting Yu

**Affiliations:** 1 Beijing Computational Science Research Center, Beijing, 100193 China; 2 Department of Physics, Zhejiang University, Hangzhou, 310027 Zhejiang China; 3 Institute of Microelectronics, Department of Microelectronics and Nanoelectronics and Tsinghua National Laboratory of Information Science and Technology, Tsinghua University, Beijing, 100084 China; 4 Department of Physics and Engineering Physics, Center for Controlled Quantum Systems, Stevens Institute of Technology, Hoboken, New Jersey 07030 USA

## Abstract

We theoretically investigate the spectral features of tunneling-induced transparency (TIT) and Autler-Townes (AT) doublet and triplet in a triple-quantum-dot system. By analyzing the eigenenergy spectrum of the system Hamiltonian, we can discriminate TIT and double TIT from AT doublet and triplet, respectively. For the resonant case, the presence of the TIT does not exhibit distinguishable anticrossing in the eigenenergy spectrum in the weak-tunneling regime, while the occurrence of double anticrossings in the strong-tunneling regime shows that the TIT evolves to the AT doublet. For the off-resonance case, the appearance of a new detuning-dependent dip in the absorption spectrum leads to double TIT behavior in the weak-tunneling regime due to no distinguished anticrossing occurring in the eigenenergy spectrum. However, in the strong-tunneling regime, a new detuning-dependent dip in the absorption spectrum results in AT triplet owing to the presence of triple anticrossings in the eigenenergy spectrum. Our results can be applied to quantum measurement and quantum-optics devices in solid systems.

## Introduction

Quantum coherence and interference effects can lead to considerably interesting phenomena of quantum optics such as lasing without inversion^1,2^, coherent population trapping^3^, correlated spontaneous emission^4^, and electromagnetically induced transparency (EIT)^5–9^. As a phenomenon closely related to EIT, Autler-Townes (AT) splitting^10,11^ is indicated by a *level anticrossing* in the eigenenergy spectrum and a transparency window owing to the AT doublet rather than the quantum interference. This phenomenon has been utilized to measure the state of the electromagnetic field^12–14^, as well as the AT triplet and multiplet spectroscopy^15^. Both EIT and AT splitting have been investigated theoretically and experimentally in different quantum systems, including atomic and molecular systems^8,16,17^, solid-state and metamaterials systems^18,19^, superconducting quantum circuits^20–26^ and whispering-gallery-mode optical resonators^27–29^. It is also interesting to investigate EIT and AT splitting in semiconductor nanostructures because the trapped carriers behave like atoms and can be conveniently manipulated via external fields.

In the semiconductor quantum dot (QD), excitons form bound states and play an important role in the optical properties of these systems^10,30–35^. Moreover, tunneling-induced transparency (TIT)^36–38^ can occur for the excitonic states, which is similar to EIT in a three-level atomic system, but no pump field is needed to apply to the excitonic system. As shown by Borges *et al*.^37^, there is an evidence regarding the coexistence of both TIT and AT doublet in the intermediate regime, when the tunneling coupling is slightly above a threshold in a double-QD system. However, a triple-QD system can offer new possibilities to study intriguing phenomena that are not observed in single and double-QD systems^39–41^. In the present paper, we show that when the electron is resonant-tunneling in such a triple-QD system, which is in contrast to the case considered in a double-QD system, the coexistence of both TIT and AT doublet regime does not occur in this system and the threshold of the tunneling coupling just corresponds to a transition point. More specifically, we find that, by analyzing the eigenenergy spectrum of the system Hamiltonian, there exist degenerate points in the eigenenergy spectrum for the resonant tunneling case. Therein the TIT presents in the weak-tunneling regime without displaying well-resolved anticrossing in eigenenergy spectrum. However, in the strong-tunneling regime, the *double anticrossings* in the eigenenergy spectrum illustrate the emergence of the AT doublet. For the off-resonance case, i.e., only the right dot is not resonant with the central dot, we show that the degenerate points are absent in the eigenenergy spectrum. The double TIT can be realized in the weak-tunneling regime along with the undistinguishable anticrossing in the eigenenergy spectrum and a new detuning-dependent transparency dip in the absorption spectrum. However, in the strong-tunneling regime, the presence of *triple anticrossings* in the eigenenergy spectrum reveals the realization of AT triplet in the absorption spectrum, where a new red-shifted (blue-shifted) transparency dip in the absorption spectrum is due to the presence of the blue (red) detuning from the right dot.

## Results

### The model

**Figure 1:**
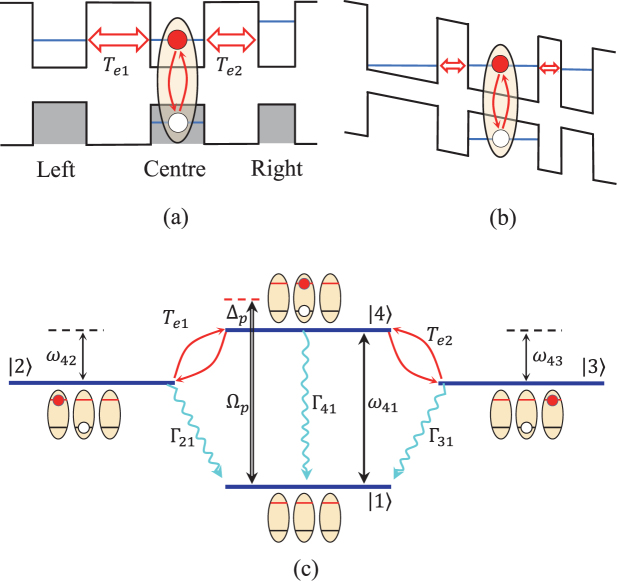
The coupled triple QD system. (**a**) Schematic energy-level diagram of a triple QD system without a gate voltage. (**b**) With an applied gate voltage, the energy-level in the conduction-band could get into resonance in the triple QD system. (**c**) Excitation scheme of the triple QD system, as determined by the Rabi frequency Ω_*p*_ (which is proportional to the probe-field strength), decoherence channels Γ_*m*1_ (*m* = 2, 3, 4), energy-level difference *ω*_4*n*_ (*n* = 1, 2, 3), probe-field detuning Δ_*p*_ = *ω*_*p*_ − *ω*_41_ from the energy-level difference *ω*_41_, and tunneling coupling *T*_*ek*_ (*k* = 1, 2). Driven by a pulsed laser field, one electron can be excited from the valence band to the conduction band to form a direct exciton state |4〉 inside the central dot. The gate electric field allows the electron to tunnel from the central dot to the left (right) dot to form an indirect excitonic states |2〉 (|3〉). Here |1〉 denotes the state with no exciton inside this triple QD.

We study a triple-QD artificial molecule consisting of three aligned QD separated by two barriers [see Fig. 1(a)]. For the purpose of inhibiting the hole tunneling between valence bands, we here consider that the central dot is identical to the left dot but structural asymmetry with the right dot. This structural asymmetry is similar to the case shown in the double quantum dots system^36,37,42–44^. With a gate (bias) voltage being applied along the growth direction, as shown in Fig. 1(b), the conduction-band-level in the left and right dot are on-resonant with the central dot^38,42,43^. In doing so, the excitonic states are composed of mostly delocalized electron states in the triple-QD accompanied with entirely localized hole states. This triple-QD system can be achieved using, e.g., a self-assembled (In, Ga) As triple-QD fabricated on a GaAs (001) substrate by molecule beam epitaxy and *in*-*situ* atomic layer precise etching, corresponding to a homogeneous triple-QD along the [1$\bar{1}$0] direction^39^. The system is driven by a weak probe laser field with frequency *ω*_*p*_ and the Rabi frequency Ω_*p*_ corresponds to the driving strength for generating the direct excitonic state in the central dot (More specifically, the electron and hole are both in the central dot). As shown in Fig. 1(c), we assume that an electron can be excited from the valence band to the conduction band via a pulsed laser field to form a direct excitonic state (denoted as |4〉) only in the central dot, where |1〉 denotes vacuum state without excitons due to the absence of optical excitation in the triple-QD system. The gate (bias) voltage just only allow the electron to tunnel from the central dot to either left or right dot, yielding an indirect excitonic state |2〉 or |3〉 in the interdot (i.e., the electron is in the central dot but the hole is in left or right dot at this moment). The Hamiltonian for this triple-QD system reads (we set *ħ* = 1)

1 $$H=\sum _{m=1}^{4}\,{\omega }_{m}{\sigma }_{mm}-({{\rm{\Omega }}}_{p}{e}^{-i{\omega }_{p}t}{\sigma }_{41}+{T}_{e1}{\sigma }_{42}+{T}_{e2}{\sigma }_{43}+{\rm{H}}.{\rm{c}}.),$$

where *σ*_*mn*_ ≡ |*m*〉 〈*n*|, and ${{\rm{\Omega }}}_{p}={\mu }_{14}{ {\mathcal E} }_{p}\mathrm{/2}\hslash$ is the Rabi frequency related to the probe laser field, with *μ*_14_ being the electric-dipole transition matrix element between |1〉 and |4〉, and ${ {\mathcal E} }_{p}$ the electric-field amplitude of the probe field. *T*_*e*1_ (*T*_*e*2_) denotes the tunneling coupling between the central dot and the left (right) dot, which can be controlled by regulating the width of the barrier and the applied gate voltage between the central dot and the left (right) dot.

Using a unitary transformation $U(t)=\exp [-i{\omega }_{p}({\sum }_{m\mathrm{=2}}^{4}\,{\sigma }_{mm})t]$ to remove the time-dependent oscillatory terms^24^, we can write the Hamiltonian in the interaction picture as

2 $${H}_{I}=-{{\rm{\Delta }}}_{p}{\sigma }_{44}-{{\rm{\Delta }}}_{2}{\sigma }_{22}-{{\rm{\Delta }}}_{3}{\sigma }_{33}-({{\rm{\Omega }}}_{p}{\sigma }_{41}+{T}_{e1}{\sigma }_{42}+{T}_{e2}{\sigma }_{43}+{\rm{H}}.{\rm{c}}.),$$

with Δ_*p*_ = *ω*_*p*_ − *ω*_41_, Δ_2_ = Δ_*p*_ + *ω*_42_, and Δ_3_ = Δ_*p*_ + *ω*_43_. Here Δ_*p*_ denotes the detuning of the probe field from *ω*_41_, and *ω*_4*n*_(*n* = 2, 3) is the energy difference between |4〉 and |*n*〉. For the triple-QD system described by Hamiltonian (2), when Δ_*p*_ = 0, the system has the degenerate dark states |*ψ*_1_〉_dark_ and |*ψ*_2_〉_dark_ as follows

3a $$|{\psi }_{1}{\rangle }_{{\rm{dark}}}=\,\cos \,\theta \mathrm{|3}\rangle -\,\sin \,\theta \mathrm{|1}\rangle ,$$

3b $$|{\psi }_{2}{\rangle }_{{\rm{dark}}}=\,\cos \,\theta \,\sin \,\varphi \mathrm{|1}\rangle +\,\sin \,\theta \,\sin \,\varphi \mathrm{|3}\rangle -\,\cos \,\varphi \mathrm{|2}\rangle ,$$

where

4 $$\tan \,\theta =\frac{{T}_{e2}}{{{\rm{\Omega }}}_{p}},\,\tan \,\varphi =\frac{{T}_{e1}}{\sqrt{{{\rm{\Omega }}}_{p}^{2}+{T}_{e2}^{2}}}.$$

Note that when *T*_*e*2_ = 0, |*ψ*_2_〉_dark_ is reduced to the dark state in a Λ-type three-level QD system, |*ψ*〉_dark_ = sin Θ|1〉 − cos Θ|2〉 with tan Θ = *T*_*e*1_/Ω_*p*_.

The dynamics of the system can be described by a Lindblad master equation:

5 $$\frac{\partial \rho }{\partial t}=-\frac{i}{\hslash }[{H}_{I},\rho ]+\sum _{m=2}^{4}\,(\frac{{{\rm{\Gamma }}}_{m1}}{2}{\mathscr{D}}[{\sigma }_{1m}]\rho +{\gamma }_{m}^{\varphi }{\mathscr{D}}[{\sigma }_{mm}]\rho ),$$

where ${\mathscr{D}}[\hat{{\mathscr{O}}}]\rho =2\hat{{\mathscr{O}}}\rho {\hat{{\mathscr{O}}}}^{\dagger }-{\hat{{\mathscr{O}}}}^{\dagger }\hat{{\mathscr{O}}}\rho -\rho {\hat{{\mathscr{O}}}}^{\dagger }\hat{{\mathscr{O}}}$, Γ_*m*1_ are the relaxation rates between |*m*〉 and |1〉, and ${\gamma }_{m}^{\varphi }$ describe the pure dephasing rates of the states |*m*〉 (*m* = 2, 3, 4). The decoherence of the excitonic states is induced by both spontaneous radiation and pure dephasing processes. For the explicit expression of the master equation, see Eq. (12) in Methods.

Also, as shown in Methods, the density matrix element *ρ*_14_ in the steady state is given by

6 $${\rho }_{14}=\frac{{d}_{2}{d}_{3}{{\rm{\Omega }}}_{p}^{\ast }}{{d}_{2}{d}_{3}{d}_{4}-{T}_{e1}^{2}{d}_{3}-{T}_{e2}^{2}{d}_{2}},$$

where *d*_2(3)_ = Δ_2(3)_ + *i*Γ_2(3)_, and *d*_4_ = Δ_*p*_ + *i*Γ_4_. It can be seen that when *T*_*e*2_ = 0, i.e., in the absence of the right-side electron tunneling in Fig. 1, Eq. (6) is reduced to the result for the linear response of a Λ-type three-level QD system^37^.

Next we decompose the density matrix element *ρ*_14_ into two components. In this way, *ρ*_14_ in Eq. (6) is decomposed as

7a $${\rho }_{14}={R}_{{\rm{I}}}+{R}_{{\rm{II}}},$$

7b $${R}_{{\rm{I}}}=\frac{{R}_{+}}{{{\rm{\Delta }}}_{p}-{{\rm{\Delta }}}_{+}},\,{R}_{{\rm{II}}}=\frac{{R}_{-}}{{{\rm{\Delta }}}_{p}-{{\rm{\Delta }}}_{-}},$$

where

8a $${{\rm{\Delta }}}_{\pm }=\frac{1}{2}[-({\omega }_{42}+i{{\rm{\Gamma }}}_{2}+i{{\rm{\Gamma }}}_{4})\pm \alpha ],\,{R}_{\pm }=\pm \frac{({{\rm{\Delta }}}_{\pm }+{\omega }_{42}+i{{\rm{\Gamma }}}_{2})}{\alpha },$$

8b $${\alpha }^{2}={[{\omega }_{42}-i({{\rm{\Gamma }}}_{4}-{{\rm{\Gamma }}}_{2})]}^{2}+4{T}_{e1}^{2}+4\frac{{d}_{2}}{{d}_{3}}{T}_{e2}^{2}.$$

The two terms *R*_I_ and *R*_II_ represent the first (“I”) and second (“II”) resonances, respectively. The two resonances can be directly used to analyze the characters of the probe-field absorption in our scheme^37,45^.

### Tunneling-induced transparency and Autler-Townes doublet

We first consider the case of an electron resonantly tunneling in the triple-QD system, i.e., *ω*_42_ = *ω*_43_ = 0. For simplicity, let Γ_2_ = Γ_3_. Then, *d*_2_ = *d*_3_, and one can analytically solve *α* = 0 in Eq. (8b) (see refs^25,29,37^ and^45^). It is shown that a transition point turns out at the threshold coupling strength ${T}_{t}\cong {{\rm{\Gamma }}}_{4}\mathrm{/2}$. Note that in the intermediate coupling regime $({T}_{t} < \sqrt{{T}_{e1}^{2}+{T}_{e2}^{2}} < {{\rm{\Gamma }}}_{4})$, in contrast to the case shown in the three-level Λ system^37^, the first-resonance *R*_I_ and the second-resonance *R*_II_, which have the same sign in absorption profile are apparently separated, indicating that there is no interference in this case. In other words, the crossover regime does not occur in this triple-QD system. Thus, there are only two regimes in such a system, i.e., the threshold *T*_*t*_ separates TIT in the weak-tunneling regime $(0 < \sqrt{{T}_{e1}^{2}+{T}_{e2}^{2}} < {T}_{t})$ from AT doublet in the strong-tunneling regime $(\sqrt{{T}_{e1}^{2}+{T}_{e2}^{2}} > {T}_{t})$.

#### (1) The weak-tunneling regime

In the weak-tunneling regime with $0 < \sqrt{{T}_{e1}^{2}+{T}_{e2}^{2}} < {T}_{t}$, *α* is a pure imaginary number. It gives rise to a pure real number ${R}_{\pm }=\mathrm{1/2}\mp {\varepsilon }_{1}/|\alpha |$, with *ε*_1_ = (Γ_4_ − Γ_2_)/2, and a pure imaginary number ${{\rm{\Delta }}}_{\pm }=i(-{\varepsilon }_{2}\pm |\alpha \mathrm{|/2)}$, with *ε*_2_ = (Γ_4_ + Γ_2_)/2. Thus, the imaginary part of *ρ*_14_ is given by

9a $${\rm{Im}}{({\rho }_{14})}_{{\rm{TIT}}}={\rm{Im}}{({R}_{{\rm{I}}})}_{{\rm{TIT}}}+{\rm{Im}}{({R}_{{\rm{II}}})}_{{\rm{TIT}}},$$

9b $${\rm{Im}}{({R}_{{\rm{I}}})}_{{\rm{TIT}}}=\frac{{C}_{{\rm{I}}}}{{{\rm{\Delta }}}_{p}^{2}+{{\rm{\Delta }}}_{+}^{2}},\,{\rm{Im}}{({R}_{{\rm{II}}})}_{{\rm{TIT}}}=\frac{-{C}_{{\rm{II}}}}{{{\rm{\Delta }}}_{p}^{2}+{{\rm{\Delta }}}_{-}^{2}},$$

where

10 $${C}_{{\rm{I}}}=(\frac{1}{2}-\frac{{\varepsilon }_{1}}{|\alpha |})\,(-{\varepsilon }_{2}+\frac{|\alpha |}{2}),\,{C}_{{\rm{II}}}=(\frac{1}{2}+\frac{{\varepsilon }_{1}}{|\alpha |})\,({\varepsilon }_{2}+\frac{|\alpha |}{2}).$$

In the weak-tunneling case, 0 < |*α*|/2 < *ε*_1_, *ε*_2_, so that both the parameters *C*_I_ and *C*_II_ are positive. Then, the first-resonance term Im(*R*_I_)_TIT_ is a wide, positive Lorentz line profile, while the second-resonance term Im(*R*_II_)_TIT_ is a narrow, negative Lorentz line profile. The different signs of the first and second resonances profile indicate the realization of the TIT, as due to the destructive interference.

**Figure 2:**
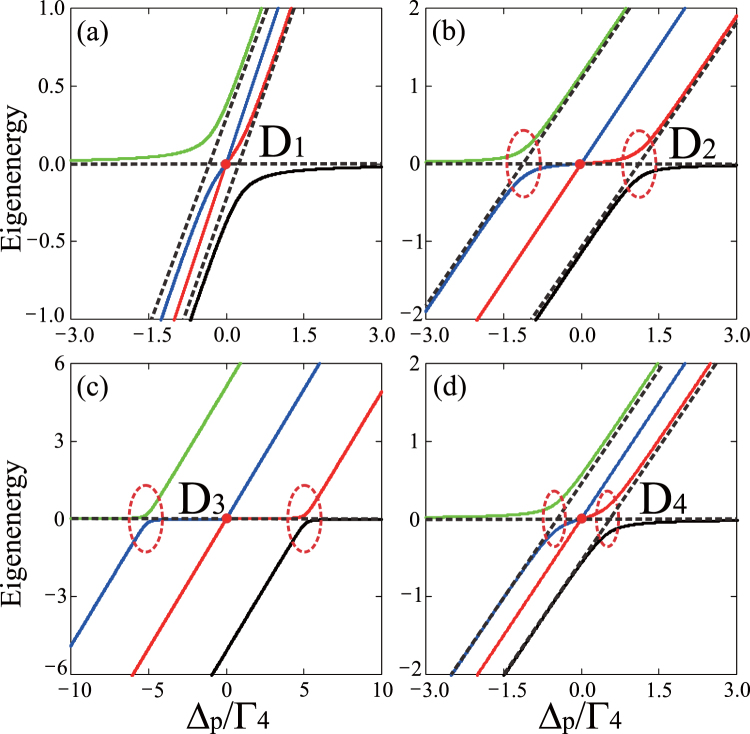
Eigenenergy spectrum of the system’s Hamiltonian with resonant detuning. Eigenenergies of the Hamiltonian (2) as a function of the probe-field detuning Δ_*p*_ at different values of the tunneling coupling strength: (**a**) *T*_*e*1_ = *T*_*e*2_ = Γ_4_/5, (**b**) *T*_*e*1_ = Γ_4_/2, *T*_*e*2_ = Γ_4_, (**c**) *T*_*e*1_ = Γ_4_, *T*_*e*2_ = 5 Γ_4_, and (**d**) *T*_*e*1_ = Γ_4_/2, *T*_*e*2_ = 10^−1^ Γ_4_, where Γ_4_ = 10 *μeV*. Other parameters are chosen as Ω_*p*_ = 10^−2^ Γ_4_ and *ω*_42_ = *ω*_43_ = 0. Here *D*_*i*_ (*i* = 1, 2, 3, 4) labels the degenerate points in the eigenenergy diagrams and the red dashed loops are used to highlight the anticrossing points.

As shown in Fig. 2(a), when the tunneling couplings *T*_*e*1_ and *T*_*e*2_ are both weak (i.e., *T*_*e*1_ = *T*_*e*2_ = Γ_4_/5), it is interesting to see that the eigenenergies of this system Hamiltonian do not display an obvious anticrossing, but show degenerate points when the electron is resonant tunneling among the triple-QD system (*ω*_42_ = *ω*_43_ = 0). However, there is an evidence of a double anticrossing in the eigenenergy spectrum of the system Hamiltonian [see the red dashed loops in Fig. 2(d)], when the relative tunneling coupling (${T}_{re}=\sqrt{{T}_{e1}^{2}+{T}_{e2}^{2}}$, with *T*_*e*1_ = Γ_4_/2, *T*_*e*2_ = 10^−1^ Γ_4_) is slightly stronger than the threshold coupling strength *T*_*t*_ = Γ_4_/2. Furthermore, these results also indicate that the coexistence of both TIT and AT doublet does not occur in such a triple-QD system, and the threshold of the tunneling coupling just corresponds to a transition point.

#### (2) The strong-tunneling regime

In the strong-tunneling regime with ${T}_{t} < \sqrt{{T}_{e1}^{2}+{T}_{e2}^{2}}$, $\alpha =2\sqrt{{T}_{e1}^{2}+{T}_{e2}^{2}}$ is a real number, such that *R*_±_ = 1/2 and Δ_±_ = −*iε*_2_ ± *α*/2. The two resonances are located at ±*α*/2 and have the same linewidth *ε*_2_. The imaginary part of *ρ*_14_ in this regime can be written as

11a $${\rm{Im}}{({\rho }_{14})}_{{\rm{ATD}}}={\rm{Im}}{({R}_{{\rm{I}}})}_{{\rm{ATD}}}+{\rm{Im}}{({R}_{{\rm{II}}})}_{{\rm{ATD}}},$$

11b $${\rm{Im}}{({R}_{{\rm{I}}})}_{{\rm{ATD}}}=\frac{-{\varepsilon }_{2}\mathrm{/2}}{{({{\rm{\Delta }}}_{p}-\frac{\alpha }{2})}^{2}+{\varepsilon }_{2}^{2}},\,{\rm{Im}}{({R}_{{\rm{II}}})}_{{\rm{ATD}}}=\frac{-{\varepsilon }_{2}\mathrm{/2}}{{({{\rm{\Delta }}}_{p}+\frac{\alpha }{2})}^{2}+{\varepsilon }_{2}^{2}}.$$

This corresponds to an AT doublet because of Im(*ρ*_14_)_ATD_ is the sum of two identical Lorentz line profile peaked at ±*α*/2^11^. It is interesting to see that the appearance of the AT doublet in the absorption spectrum of the probe field is equivalent to the case that there exist the double anticrossings in the eigenenergies of the system Hamiltonian [as shown in the red dashed loops in Fig. 2(b)], forming a transparency window between the pair of resonances. Now the positive value of the resonance pair is only responsible for the decreasing or even vanishing absorption of the probe field, instead of accounting for this phenomenon through the cause for that of the destructive interference in the TIT^29,37^. This is because the pair of resonances is shifted relatively far away from each other, so that their overlap is insufficient to yield significant interference.

When the tunneling couplings are sufficiently strong (*T*_*e*1_, *T*_*e*2_ ≥ Γ_4_), there are prominent double anticrossings in the eigenenergies of the system Hamiltonian [as shown in the red dashed loops in Fig. 2(c)]. This also gives rise to an evident reduction of the overall absorption, displaying a wide transparency window (corresponding to vanishing absorption)^37^. The pronounced double anticrossings in this situation can be used to deduce the positions and width of the transparency window of the AT doublet. Therefore, in this strong-tunneling regime, the TIT evolves to the AT doublet, which results in a well-resolved doublet in the absorption spectrum and double anticrossings in the eigenenergy spectrum.

### Double Tunneling-induced transparency and Autler-Townes triplet

**Figure 3:**
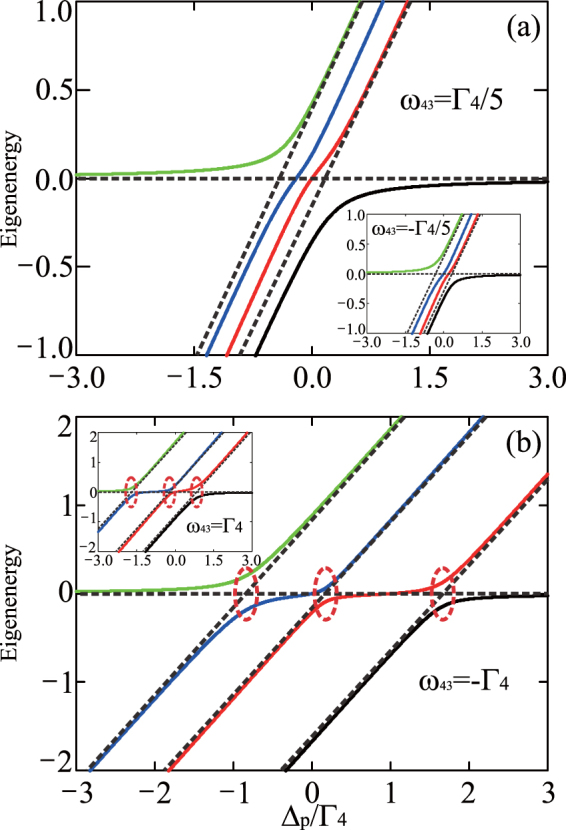
Eigenenergy spectrum of the system’s Hamiltonian with off-resonant detuning. Eigenenergies of the Hamiltonian (2) as a function of the probe-field detuning Δ_*p*_ at different values of the tunneling couplings strength and the frequency difference *ω*_43_: (**a**) *T*_*e*1_ = *T*_*e*2_ = Γ_4_/5, *ω*_43_ = ±Γ_4_/5, and (**b**) *T*_*e*2_ = 2*T*_*e*1_ = Γ_4_ = 10 *μeV*, *ω*_43_ = ±Γ_4_, with *ω*_42_ = 0 and Ω_*p*_ = 10^−2^ Γ_4_. The red dashed loops are used to highlight the anticrossing points.

Next, we consider the case of an electron resonant tunneling between the left and central dots (i.e., *ω*_42_ = 0), while there is off-resonant tunneling between the right and central dots (that is, *ω*_43_ ≠ 0) in the triple-QD system. The energy shift induced by the gate field is given by Δ*ω*_43_ = *eFd*^38,42^, with *F* being the fixed electric field and *d* being the width of the barrier between the right and central dot. In this scenario, the optical absorption of the probe field can be manipulated by varying the fixed electric field *F*, which is a way of controlling the detuning of the right dot *ω*_43_. It should be noted that, as shown in Fig. 3, the degenerate points of the eigenenergies of the system Hamiltonian disappear when *ω*_43_ is nonzero. For instance, when *ω*_43_ = ±Γ_4_/5, there are not degenerate points and distinguishable anticrossing in the eigenenergies of the system Hamiltonian in the weak-tunneling regime [see Fig. 3(a)], which is clearly distinct from the results shown in a double-QD system^37^. In the strong-tunneling regime, one can also see that the eigenenergies of the system Hamiltonian exhibit triple anticrossings [see Fig. 3(b)], as compared to the case with double anticrossings being implied to denote AT doublet in Fig. 2(b) where *ω*_43_ = 0.

#### (1) The weak-tunneling regime with *ω*_43_ ≠ 0

**Figure 4:**
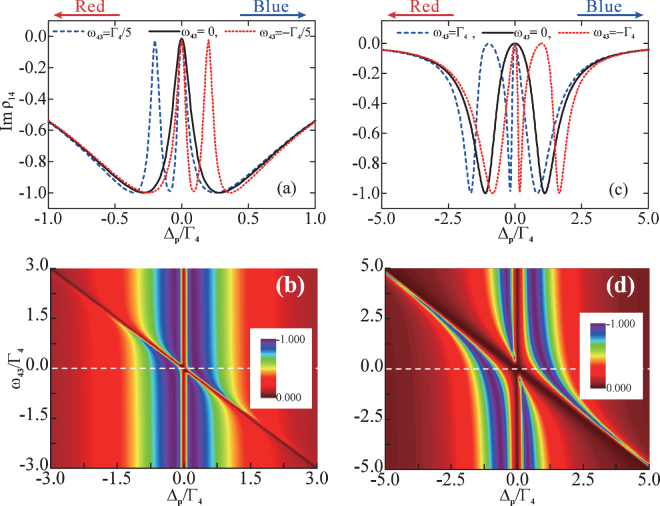
Double Tunneling-induced transparency and Autler-Townes triplet. (**a**,**b**) Im(*ρ*_14_) as a function of the probe-field detuning Δ_*p*_ at different values of the frequency difference *ω*_43_, where *T*_*e*1_ = *T*_*e*2_ = Γ_4_/5; (**c**,**d**) Im(*ρ*_14_) as a function of both the probe-field detuning Δ_*p*_ and the frequency difference *ω*_43_, where *T*_*e*2_ = 2*T*_*e*1_ = Γ_4_ = 10 *μeV*. Here *ω*_42_ = 0 and Γ_2_ = Γ_3_ = 10^−3^ Γ_4_.

As shown in Fig. 4(a), in the weak-tunneling regime, it is revealed that double TIT can be realized by manipulating the energy-level detuning Δ_3_ to achieve slight off-resonance. Narrow double transparency windows arise, when the tunneling couplings are weaker than or equal to the threshold value ${T^{\prime} }_{t}$ [as shown in Eq. (8b), there is indeed threshold value in this case but no explicit expression], in the case of *ω*_43_ ≠ 0. In particular, the new TIT dip [see the blue dashed curve in Fig. 4(a)] is red-shifted for a blue-detuned Δ_3_ (e.g., *ω*_43_ = Γ_4_/5) in the probe field absorption spectrum. However, the new TIT dip [see the red dotted curve in Fig. 4(a)] becomes blue-shifted at a red-detuned Δ_3_ (e.g., *ω*_43_ = −Γ_4_/5) in the absorption spectrum. Furthermore, both the undistinguished anticrossing in the eigenenergy spectrum [see Fig. 3(a)] and the new detuning-dependent dip presenting within the scope of the full-linewidth [see the black curve in Fig. 4(a)] in the absorption spectrum demonstrate that the double TIT is implemented. Hence, the double TIT is significantly different from AT doublet, where the peaks of the pair resonances are far enough separated apart and the double anticrossings occur in the eigenenergy spectrum. Moreover, as shown in Fig. 4(b), one of the absorption minima in the probe field absorption spectrum obeys the condition Δ_*p*_ = *ω*_42_ = 0, and the other absorption minimum satisfies the condition Δ_*p*_ = −*ω*_43_. Therefore, we are able to realize double TIT without forming well-resolved anticrossing in such a weak-tunneling regime.

#### (2) The strong-tunneling regime with *ω*_43_ ≠ 0

In the strong-tunneling regime, the transparency window of the AT doublet [see the black solid curve in Fig. 4(c)] exhibits a new peak, turning the probe field absorption profile into three peaks. This yields two transparency windows [as shown in the blue dashed and red dotted curves in Fig. 4(c)], where a new dip arises in the blue (red) side of the probe detuning accompanied with the other two peaks of the transparency window being blue-shifted (red-shifted) when there is a red-detuned (blue-detuned) Δ_3_, e.g., ${\omega }_{43}=\mp {{\rm{\Gamma }}}_{4}\mathrm{/5}$. Also, one can see that the triple anticrossings in the eigenenergy spectrum [see Fig. 3(b)] make sure that the AT triplet is fulfilled at this moment. In Fig. 4(d), similar features can be observed, where two absorption minima in the absorption spectrum locate at Δ_*p*_ = *ω*_42_ = 0 and Δ_*p*_ = −*ω*_43_, respectively. It should be noted that the width of the central peak increases when raising the blue-detuning (red-detuning) Δ_3_, but the width of the peak on the red-detuned (blue-detuned) side decreases. In particular, for a blue-detuned (red-detuned) Δ_3_, the decrease of the width of the red-detuned (blue-detuned) sideband is compensated by the increase of the width of the central peak. Therefore, in the strong-tunneling regime, the AT triplet can be realized by manipulating the detuning Δ_3_.

## Discussion

In this work, we have presented a theoretical study of the eigenenergy spectrum and optical absorption properties of a triple-QD system with four effective energy levels. The results show that, in the case of the electron resonant-tunneling in the triple-QD system, there are degenerate points in the eigenenergy spectrum of the system Hamiltonian. In the weak-tunneling regime, the presence of the TIT does not show an obvious anticrossing in the eigenenergy spectrum. However, in the strong-tunneling regime, the emergence of double anticrossings in the eigenenergy spectrum indicates that the TIT evolves to the AT doublet which includes two well-resolved peaks in the probe field absorption spectrum. The pronounced double anticrossings in the eigenenergy spectrum of the system Hamiltonian that can be used to deduce the positions and width of the transparency window of the AT doublet. For the off-resonance case, that is, the right dot is not resonant with the central dot, we demonstrate that the degenerate points in the eigenenergy spectrum disappear. The realization of double TIT in the weak-tunneling regime, where the distinguishable anticrossing does not appear in the eigenenergy spectrum, exhibits a new transparency dip in the absorption spectrum which can be controlled by manipulating one of the energy-level detunings. However, in the strong-tunneling regime, the presence of triple anticrossings in the eigenenergy spectrum illustrates the realization of AT triplet in the absorption spectrum. More importantly, there is a new dip in the blue (red) side of the probe detuning along with the other two peaks of the transparency window being blue-shifted (red-shifted) at a red-detuned (blue-detuned) Δ_3_. The linewidth narrowing in one of the side peaks could be compensated for the linewidth broadening in the central peak.

Finally, our proposed schemes for these spectral features are naturally inherited and open up new ways for physicists and chemists to work in the field of laser spectroscopy, quantum measurement, nonlinear optics and quantum-optics devices in solid systems.

## Methods

### Quantum dynamics behavior of the triple QD system

By applying the Born-Markov approximation, the coupled differential equations for the density matrix *ρ*_*mn*_ in the interaction picture can be obtained as

12 $$\begin{array}{rcl}{\partial }_{t}{\rho }_{11} & = & {{\rm{\Gamma }}}_{21}{\rho }_{22}+{{\rm{\Gamma }}}_{31}{\rho }_{33}+{{\rm{\Gamma }}}_{41}{\rho }_{44}-i{{\rm{\Omega }}}_{p}{\rho }_{14}+i{{\rm{\Omega }}}_{p}^{\ast }{\rho }_{41},\\ {\partial }_{t}{\rho }_{22} & = & -{{\rm{\Gamma }}}_{21}{\rho }_{22}-i{T}_{e1}{\rho }_{24}+i{T}_{e1}{\rho }_{42},\\ {\partial }_{t}{\rho }_{33} & = & -{{\rm{\Gamma }}}_{31}{\rho }_{33}-i{T}_{e2}{\rho }_{34}+i{T}_{e2}{\rho }_{43},\\ {\partial }_{t}{\rho }_{44} & = & -{{\rm{\Gamma }}}_{41}{\rho }_{44}+i{{\rm{\Omega }}}_{p}^{\ast }{\rho }_{14}+i{T}_{e1}{\rho }_{24}+i{T}_{e2}{\rho }_{34}\\  &  & -i{{\rm{\Omega }}}_{p}{\rho }_{41}-i{T}_{e1}{\rho }_{42}-i{T}_{e2}{\rho }_{43},\\ {\partial }_{t}{\rho }_{12} & = & i({{\rm{\Delta }}}_{2}+i{{\rm{\Gamma }}}_{2}){\rho }_{12}-i{T}_{e1}{\rho }_{14}+i{{\rm{\Omega }}}_{p}^{\ast }{\rho }_{42},\\ {\partial }_{t}{\rho }_{13} & = & i({{\rm{\Delta }}}_{3}+i{{\rm{\Gamma }}}_{3}){\rho }_{13}-i{T}_{e2}{\rho }_{14}+i{{\rm{\Omega }}}_{p}^{\ast }{\rho }_{43},\\ {\partial }_{t}{\rho }_{14} & = & i({{\rm{\Delta }}}_{p}+i{{\rm{\Gamma }}}_{4}){\rho }_{14}-i{T}_{e1}{\rho }_{12}-i{T}_{e2}{\rho }_{13}\\  &  & -i{{\rm{\Omega }}}_{p}^{\ast }({\rho }_{11}-{\rho }_{44}),\\ {\partial }_{t}{\rho }_{23} & = & i({{\rm{\Delta }}}_{3}-{{\rm{\Delta }}}_{2}+i{\gamma }_{23}){\rho }_{23}-i{T}_{e2}{\rho }_{24}+i{T}_{e1}{\rho }_{43},\\ {\partial }_{t}{\rho }_{24} & = & i({{\rm{\Delta }}}_{p}-{{\rm{\Delta }}}_{2}+i{\gamma }_{24}){\rho }_{24}-i{{\rm{\Omega }}}_{p}^{\ast }{\rho }_{21}-i{T}_{e2}{\rho }_{23}\\  &  & -i{T}_{e1}({\rho }_{22}-{\rho }_{44}),\\ {\partial }_{t}{\rho }_{34} & = & i({{\rm{\Delta }}}_{p}-{{\rm{\Delta }}}_{3}+i{\gamma }_{34}){\rho }_{34}-i{{\rm{\Omega }}}_{p}^{\ast }{\rho }_{31}-i{T}_{e1}{\rho }_{32}\\  &  & -i{T}_{e2}({\rho }_{33}-{\rho }_{44}),\end{array}$$

with ${{\rm{\Gamma }}}_{m}={{\rm{\Gamma }}}_{m1}\mathrm{/2}+{\gamma }_{m}^{\varphi }$ (*m* = 2, 3 and 4), and ${\gamma }_{mn}=({{\rm{\Gamma }}}_{m1}+{{\rm{\Gamma }}}_{n1}\mathrm{)/2}+{\gamma }_{m}^{\varphi }+{\gamma }_{n}^{\varphi }$ (*m* = 2, 3; *n* = 3, 4).

### The derivation of the solution of the density matrix *ρ*_14_

From Eq. (12), when the time is explicitly shown in the equations of ∂_*t*_*ρ*_12_, ∂_*t*_*ρ*_13_, and ∂_*t*_*ρ*_14_, it follows that

13 $$\begin{array}{rcl}{\partial }_{t}{\rho }_{12}(t) & = & i({{\rm{\Delta }}}_{2}+i{{\rm{\Gamma }}}_{2}){\rho }_{12}(t)-i{T}_{e1}{\rho }_{14}(t)+i{{\rm{\Omega }}}_{p}^{\ast }{\rho }_{42}(t),\\ {\partial }_{t}{\rho }_{13}(t) & = & i({{\rm{\Delta }}}_{3}+i{{\rm{\Gamma }}}_{3}){\rho }_{13}(t)-i{T}_{e2}{\rho }_{14}(t)+i{{\rm{\Omega }}}_{p}^{\ast }{\rho }_{43}(t),\\ {\partial }_{t}{\rho }_{14}(t) & = & i({{\rm{\Delta }}}_{p}+i{{\rm{\Gamma }}}_{4}){\rho }_{14}(t)-i{T}_{e1}{\rho }_{12}-i{T}_{e2}{\rho }_{13}(t)\\  &  & -i{{\rm{\Omega }}}_{p}^{\ast }({\rho }_{11}(t)-{\rho }_{44}(t\mathrm{))}.\end{array}$$

The absorption and dispersion coefficients are respectively proportional to the imaginary and real parts of the density matrix element *ρ*_14_ in the steady state^37,45^. With a weak probe field (${{\rm{\Omega }}}_{p}\ll {T}_{e1},{T}_{e2}$) acting on the considered triple-QD system, the term ${{\rm{\Omega }}}_{p}^{\ast }{\rho }_{42}(t)$ and ${{\rm{\Omega }}}_{p}^{\ast }{\rho }_{43}(t)$ in Eq. (13) can be respectively approximated by ${{\rm{\Omega }}}_{p}^{\ast }{\rho }_{42}\mathrm{(0)}$ and ${{\rm{\Omega }}}_{p}^{\ast }{\rho }_{43}\mathrm{(0)}$, and the term ${{\rm{\Omega }}}_{p}^{\ast }[{\rho }_{11}(t)-{\rho }_{44}(t)]$ can also be approximated by ${{\rm{\Omega }}}_{p}^{\ast }[{\rho }_{11}\mathrm{(0)}-{\rho }_{44}\mathrm{(0)]}$. Moreover, the system is assumed to be initially in the ground state |1〉, so *ρ*_11_(0) = 1 and *ρ*_42_(0) = *ρ*_43_(0) = *ρ*_44_(0) = 0. Then, Eq. (13) becomes

14 $$\begin{array}{rcl}{\partial }_{t}{\rho }_{12} & = & i({{\rm{\Delta }}}_{2}+i{{\rm{\Gamma }}}_{2}){\rho }_{12}-i{T}_{e1}{\rho }_{14},\\ {\partial }_{t}{\rho }_{13} & = & i({{\rm{\Delta }}}_{3}+i{{\rm{\Gamma }}}_{3}){\rho }_{13}-i{T}_{e2}{\rho }_{14},\\ {\partial }_{t}{\rho }_{14} & = & i({{\rm{\Delta }}}_{p}+i{{\rm{\Gamma }}}_{4}){\rho }_{14}-i{T}_{e1}{\rho }_{12}-i{T}_{e2}{\rho }_{13}-i{{\rm{\Omega }}}_{p}^{\ast }.\end{array}$$

In the steady-state, ∂_*t*_*ρ*_12_ = ∂_*t*_*ρ*_13_ = ∂_*t*_*ρ*_14_ = 0, so we have

15 $${\rho }_{14}=\frac{{d}_{2}{d}_{3}{{\rm{\Omega }}}_{p}^{\ast }}{{d}_{2}{d}_{3}{d}_{4}-{T}_{e1}^{2}{d}_{3}-{T}_{e2}^{2}{d}_{2}}.$$
